# Neural activation in photosensitive brain regions of Atlantic salmon (*Salmo salar*) after light stimulation

**DOI:** 10.1371/journal.pone.0258007

**Published:** 2021-09-29

**Authors:** Mariann Eilertsen, Benjamin G. J. Clokie, Lars O. E. Ebbesson, Cristina Tanase, Herve Migaud, Jon Vidar Helvik

**Affiliations:** 1 Department of Biological Sciences, University of Bergen, Bergen, Norway; 2 Institute of Aquaculture, University of Stirling, Stirling, Scotland, United Kingdom; 3 Norce, Bergen, Norway; 4 Signify, Eindhoven, Netherlands; Universidade de Vigo, SPAIN

## Abstract

Photoreceptive inputs to the teleost brain are perceived as image of the visual world and as photo-modulation of neuroendocrine and neuronal signals. The retina and pineal organ are major receptive organs with projections to various parts of the brain, but in the past decades deep brain photoreceptors have emerged as candidates for photoreceptive inputs, either independent or in combination with projections from light sensory organs. This study aimed to test the effects of narrow bandwidth light using light-emitting diodes technology on brain neural activity through putative opsin stimulation in Atlantic salmon. The expression of *c-fos*, a known marker of neural activity, was compared *in situ* between dark-adapted salmon parr and following light stimulation with different wavelengths. *c-fos* expression increased with duration of light stimulation and the strongest signal was obtained in fish exposed to light for 120 minutes. Distinct and specific brain regions were activated following dark to light stimulation, such as the habenula, suprachiasmatic nucleus, thalamus, and hypothalamus. The *c-fos* expression was overlapping with photoreceptors expressing melanopsin and/or vertebrate ancient opsin, suggesting a potential direct activation by light. Interestingly in the habenula, a distinct ring of vertebrate ancient opsin and melanopsin expressing cells is overlapping with *c-fos* expression after neural activation. Salmon exposed to different spectra had neural activation in similar brain regions. The most apparent difference was melanopsin expression in the lateral cells of the lateral tuberal nuclus in the hypothalamus, which appeared to be specifically activated by red light. Light-stimulated neuronal activity in the deep brain was limited to subpopulations of neurons, mainly in regions with neuronal modulation activity, retinal and pineal innervations and known presence of nonvisual photoreceptors. The overlapping expression patterns of *c-fos* and nonvisual opsins support direct light stimulation of deep brain photoreceptors and the importance of these systems in light induced brain activity.

## Introduction

Light relays important cues for regulating biological processes and rhythms in animals. Photoreception in teleosts is often related to sensory organs such as the retina and pineal which have innervations to several regions of the brain such as the optic tectum, preoptic area, habenula, thalamus, hypothalamus and tegmentum [1]. However, in the last decade new photoreceptor families have been identified with central expression (e.g., the deep brain) rather than peripheral [2–5]. Studies have shown that the fish brain is packed with photoreceptor elements [2, 4, 6–12], but knowledge about activation of these photoreceptor systems and the regulation of biological processes is limited.

In the past years, the nonvisual opsins have been shown to have specific photosensitive functions in the teleost brain. Zebrafish (*Danio rerio*) larvae lacking eyes and pineal were shown to display light seeking behavior following exposure to darkness regulated by melanopsin-expressing cells in the preoptic area [8]. Our previous studies have shown that a transient cluster of dual photoreceptors, expressing vertebrate ancient opsin (VA opsin) and melanopsin, in the hindbrain are permissive to the life history transition of hatching in Atlantic halibut (*Hippoglossus hippoglossus)* embryo with neuroblastic retina [13]. In masu salmon (*Oncorhynchus masou*), the saccus vasculosus, expressing e.g., short wavelength-sensitive opsin and melanopsin, has been indicated to act as a sensor of seasonal changes in day-length in fish [14] however, the suggestion is debated [15]. Activation of photoreceptors in the brain after light stimulation can be visualized by expression studies of *c-fos*, known to respond rapidly and transiently to a variety of stimuli such as growth factor stimulation and stimulation of nerve cells [16, 17]. Studies analyzing the neural activity following stimulation with a light pulse in both mammals [18, 19] and fish [13, 20] showed increased expression levels of *c-fos*. Zebrafish was shown to have a strong response to changing photic conditions, as light-dark transition, and a 30-minute light pulse at night induced *c-fos* expression in brain regions co-localised with deep brain photoreceptors. The study suggested that elevated level of *c-fos* expression would reflect a distinct change in neural activity in response to changes in sensory input [20]. Further, our studies in Atlantic halibut showed *c-fos* activation in the dual photoreceptive hindbrain cluster and hatching glands after light induced hatching. The hindbrain cluster was shown to be imbedded in a neuronal network projecting to the narrow belt of hatching glands in the yolk sac, indicating a direct light control of hatching by neuronal activation [13].

In Atlantic salmon (*Salmo salar*), the changing seasonal photoperiod strongly influences the physiology and behavior throughout the lifecycle. Photoperiod regulates development and growth, timing of smoltification, migration and maturation [21–26]. Salmon is therefore an interesting species to study the underlying mechanisms of photo-induced brain activity. Narrow bandwidth lights using light-emitting diodes (LED) technology have been used to explore the effects of light spectra on development, growth and survival in a range of species including zebrafish [27], Atlantic cod (*Gadus morhua*) and turbot (*Scophthalmus maximus*) [28]. The present study aimed to determine the effects of narrow bandwidth light on activation of photoreceptive brain regions by using *c-fos* as a marker of neural activity. To do so, Atlantic salmon parr were exposed to white light and narrow bandwidth light using LED- technology, to explore brain light sensitivity and reveal brain regions that are activated following light stimulation. Our results revealed distinct neural activity after 120 minutes light stimulation in several putative photoreceptive regions of the brain also having retinal and pineal innervations, such as the habenula, suprachiasmatic nucleus, thalamus, and hypothalamus. Salmon exposed to different spectra had neural activation in similar brain regions, however we found melanopsin expressing lateral cells of the lateral tuberal nucleus of hypothalamus which specifically appeared to be activated by red light.

## Materials and methods

### Animals and sampling

Juvenile Atlantic salmon (*Salmo salar*) parr (freshwater) (approx. 13 cm) were sourced from ILAB (Industry Laboratory), Bergen, Norway to perform the initial *c-fos* activation study to determine the optimal stimulation time (Activation time response of *c-fos*). The use and handling of animals was performed according to Norwegian law and the Norwegian Animal Research Authority (NARA) following procedures at the authorized facility and was given ethical approval by the Norwegian Veterinary Authorities (Application number: 6918). Atlantic salmon parr (approx. 19 cm) used for the narrow bandwidth experiment in Stirling were sourced from the Niall Bromage Freshwater Research Facility (Institute of Aquaculture, University of Stirling, Stirling). The trial was carried out at the University of Stirling temperate freshwater facilities with all experimental procedures conducted in compliance with the Animals Scientific Procedures Act 1986 (Home Office Code of Practice. HMSO: London January 1997) under project license PPL70/7916 “Environmental Regulation of Fish Physiology” in accordance with EU regulation (EC Directive 86/609/EEC). All experimentation performed at the Institute of Aquaculture was subject to an ethical review process carried out by the University of Stirling Animal Welfare and Ethical Review Board prior to the work being conducted. All fishes were euthanized with an overdose of methacaine (MS-222, Sigma, USA) before perfusion fixation.

### Activation time response of *c-fos*

To identify the optimal sampling point after stimulation with light, for the subsequent light activation study using *in situ* hybridization, a preliminary test sampling was performed. Atlantic salmon parr were acclimatized in tanks for 5 days before being subjected to darkness for 48 hours. Fish were then stimulated for 15, 30, 60 or 120 minutes with two wide spectrum compact fluorescent bulbs (Viva-Lite, Germany). Sampling for *in situ* hybridization consisted of five fish per treatment (duration of stimulation) euthanized with an overdose of methacaine and fixated by vascular perfusion through the heart with 4% paraformaldehyde. In addition, a dark control (5 fish) was included. The brains were dissected out and prepared for *in situ* hybridization on cryo-sections as described by [12]. The ISH was carried out under similar conditions for all sampling points.

### Narrow bandwidth light activation experiment

**Fig 1:**
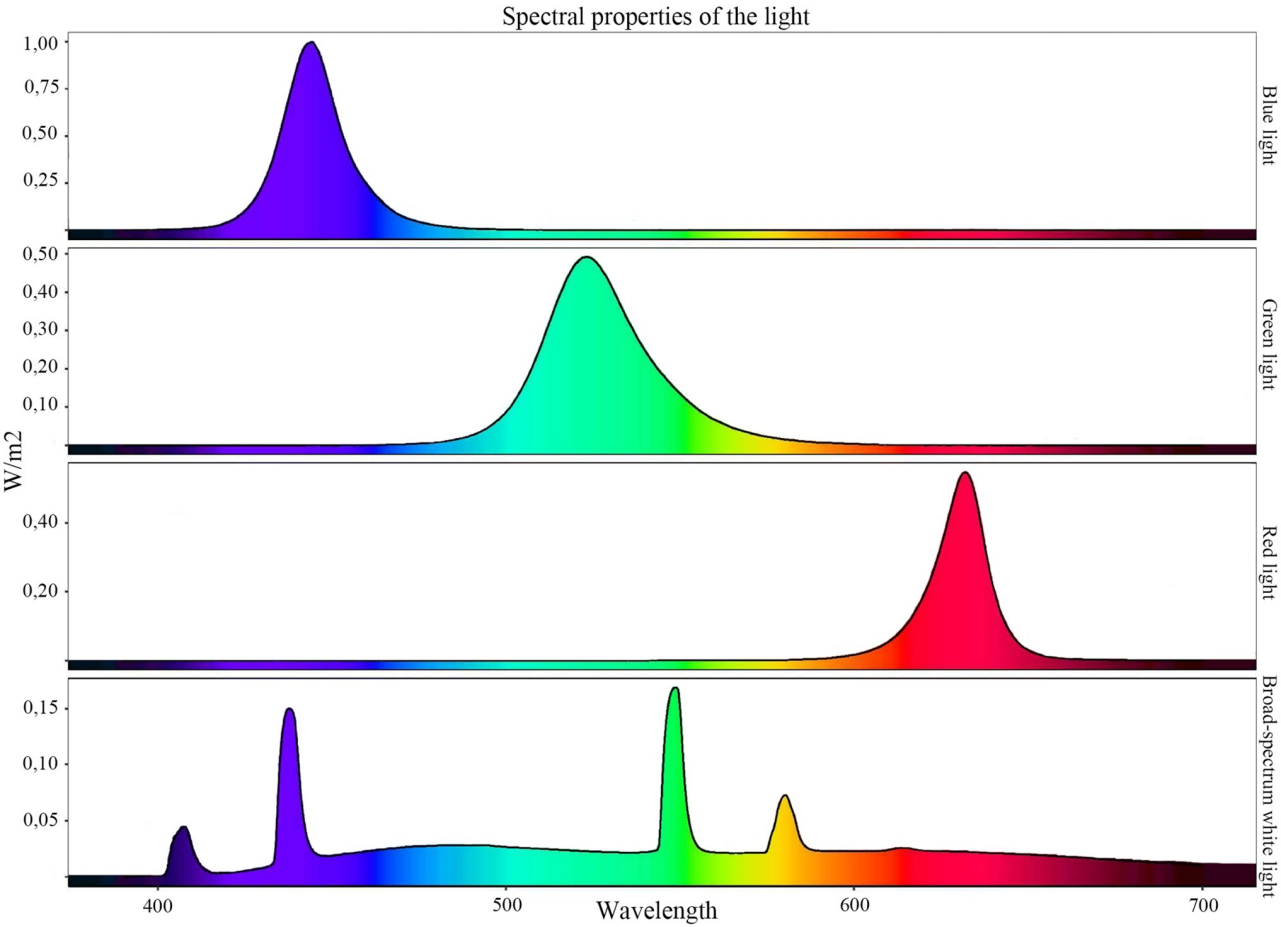
Spectral composition of the experimental light. LED units (Signify) generated narrow bandwidth lights of Blue (444 nm peak), Green (523 nm peak) or Red (632 nm peak). Broad-spectrum white light was delivered using a wide spectrum compact fluorescent bulb (Viva-Lite). Intensity in each tank for all light treatments was calibrated to 5 W/m^2^.

Atlantic salmon (parr) maintained under constant light since hatching were transferred into a recirculating aquaculture system (University of Stirling, UK) and acclimatized in tanks for 5 days before being exposed to darkness for 48 hours. The experiment included thirteen experimental groups held in separate tanks including controls. The experiment was divided in an on-response (dark to light) and an off-response (light to dark). In dark to light groups, fish were exposed to different narrow bandwidth light (Dark/White, Dark/Blue, Dark/Green, Dark/Red and Dark/Dark as the control) for 120 minutes before sampling. In the light to dark groups, dark-adapted fish were exposed to different narrow bandwidth light for 24 hours before returning them to darkness for 120 min (White/Dark, Blue/Dark, Green/Dark, Red/Dark) or not (control) (White/White, Blue/Blue, Green/Green, Red/Red) and then sampling. At each sampling, fish were euthanized and perfused as described above. Narrow bandwidth lights were supplied by Signify using computer-controlled LED units, producing either Blue (444 nm peak), Green (523 nm peak) or Red (632 nm peak) light. The three narrow bandwidth lights were chosen to span different parts of the broad-spectrum white light. The broad-spectrum white light was chosen since it had a broad absorbance spectrum within the range of visible light. Broad-spectrum white light provided for both the test sampling and narrow bandwidth light activation experiment was delivered using two wide spectrum compact fluorescent bulb (Viva-Lite, Germany). Light intensity was measured just below the water surface directly below the lamp using a single sensor channel Watts’s meter (Skye Instruments Ltd, UK) calibrated to National Physics Laboratory (UK) Standards. Intensity in each tank for all light treatments was calibrated to 5 W/m^2^. See Fig 1 for spectral composition of the experimental light.

### Identification of *c-fos* paralogue genes

The salmon database Ssa_ASM_3.6.scaf.fasta at ViroBLAST (http://marineseq.imr.no/salmon2014/viroblast/viroblast.php) was searched with a query sequence of Atlantic halibut *c-fos* (Accession number: KF941297) giving two relevant hits (ccf1000000124_0–0 and ccf1000000152_0–0). Alignment of the query sequence and subject (contig) indicated two potential *c-fos* genes. Based on the alignment the exons were predicted and primers located in the potential UTR’s were designed for the two genes. Primers are listed in Table 1.

**Table 1:** Primers for molecular cloning.

Primer name	Sequence (5’–3’)
cfos124F1	GGATCACTTGACTTTGACAGC
cfos124R1	TGCGCTGAAGAACAAGTCAAC
cfos152F1	GGGATCACTTGACTTTGACAAC
cfos152R1	GCTTCCTGGTTGTGCGAGTC

Primers used for cloning of the two *c-fos* paralogues.

### Molecular cloning

Total RNA isolation, DNase treatment and cDNA synthesis were performed according to [29]. The two Atlantic salmon *c-fos* genes were cloned by amplification of the genes by PCR using a pool of DNase treated cDNA of Atlantic salmon brains. PCR was run with annealing temperature of 63°C for *c-fos*.*1* and 62°C for *c-fos*.*2* and 35 cycles were used. The primer pairs for both genes generated a PCR product of approximately 1500 bp. The PCR products were separated on an agarose gel and the relevant bands were cut out and extracted from the agarose gel by QIAEX II Gel Extraction kit (Qiagen, Germany). The PCR products were ligated into StrataClone PCR cloning vector pSSC-A-amp/kan (Agilent Technologies, USA) and sequenced at the University of Bergen Sequencing Facility. The nucleotide sequences were deposited into GeneBank with the accession number MF685241 for *c-fos*.*1* (ccf1000000124_0–0) and MF685242 for *c-fos*.*2* (ccf1000000152_0–0).

### Synthesis of RNA probe for *c-fos*, melanopsin and VA opsin

For the synthesis of RNA probes, PCR product was used as template for the reaction and primers were designed for *c-fos*, melanopsin and VA opsin as described by [30]. The primers for *c-fos* were designed to be specific for both paralogues by making them degenerative, ensuring a probe specific for both genes. For melanopsin, three probes were synthesized and used together, *opn4m1a1* (specific for JN210547), *opn4x1a* (specific for JN210546) and *opn4x1b1/2* (specific for both JN210544 and JN210545) ensuring to detect all the functional photopigments of melanopsin. For VA opsin the probe primer was designed based on NM_001123626. Synthesis of DIG-labelled RNA probe for *c-fos* and the melanopsins and DIG and FITC-labelled RNA probe for VA opsin was done following the manufacturer’s instructions (Roche Diagnostics, Germany). The synthesized probes were precipitated by LiCl and EtOH together with tRNA (Roche Diagnostics, Germany).

### *In situ* hybridization and Nissl staining

Parallel sectioning (10μm) was performed on a Leica CM 3050S cryostat (Leica Biosystems, Germany) and before storage at 20°C, the tissues were air dried for 1 hour at room temperature and for 30 minutes at 65°C. *In situ* hybridization (ISH) was carried out as described by [12]. One parallel of the sectioned brain was Nissl-stained with 0.5% cresyl fast violet (Chroma-Gesellschaft, Germany), and the other parallel was stained by *in situ* hybridization.

## Results

### Activation time response of *c-fos*

**Fig 2:**
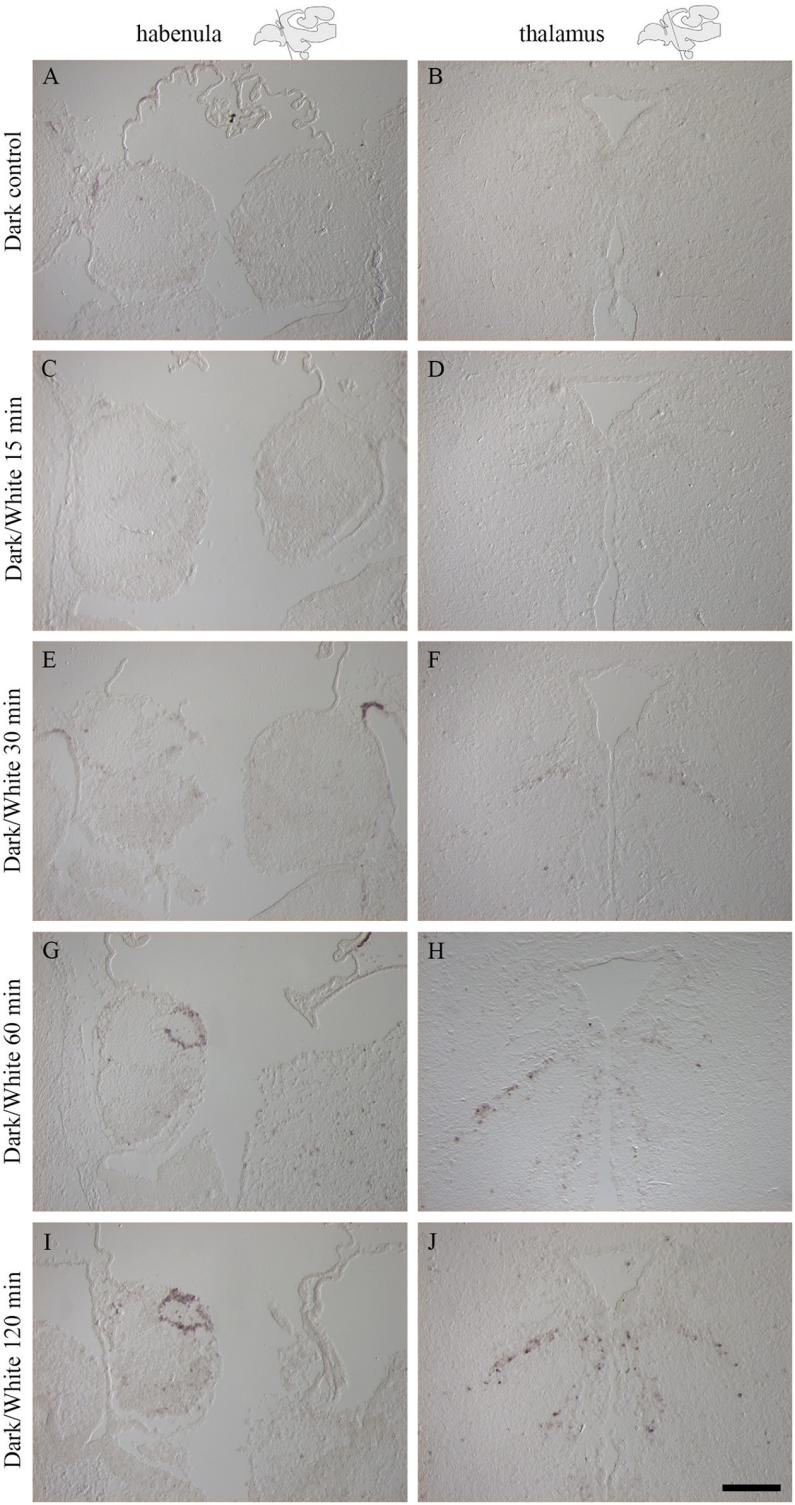
Activation of the immediate early gene *c-fos* after stimulation with white light was strongest after 120 minutes of light exposure. *In situ* hybridization with *c-fos* is shown in the habenula (A, C, E, G, I) and thalamus (B, D, F, H, J) and schematic drawings indicate the plane of the cryo-section. A-B: Dark control, no *c-fos* expression detected. C-D: Sampling after 15 minutes exposure to white light, no *c-fos* expression detected. E-F: Sampling after 30 minutes exposure, weak *c-fos* expression detected. G-H: Sampling after 60 minutes exposure, strong *c-fos* expression detected. I-J: Sampling after 120 minutes exposure, the strongest *c-fos* expression detected. Scale bar of 200 μm.

Fish kept in darkness showed no detectable expression of *c-fos* (Fig 2A and 2B). Stimulation with light for 15 minutes did not result in any detectable *c-fos* expression by ISH (Fig 2C and 2D). Expression levels of *c-fos* increased with the duration of the light stimulation (detectable but weak after 30 minutes (Fig 2E and 2F), enhanced after 60 minutes (Fig 2G and 2H) and very strong after 120 minutes (Fig 2I and 2J)). A light stimulation duration of 120 minutes was therefore selected for subsequent experiment and *c-fos* ISH analyses.

### White light neural activation

**Fig 3:**
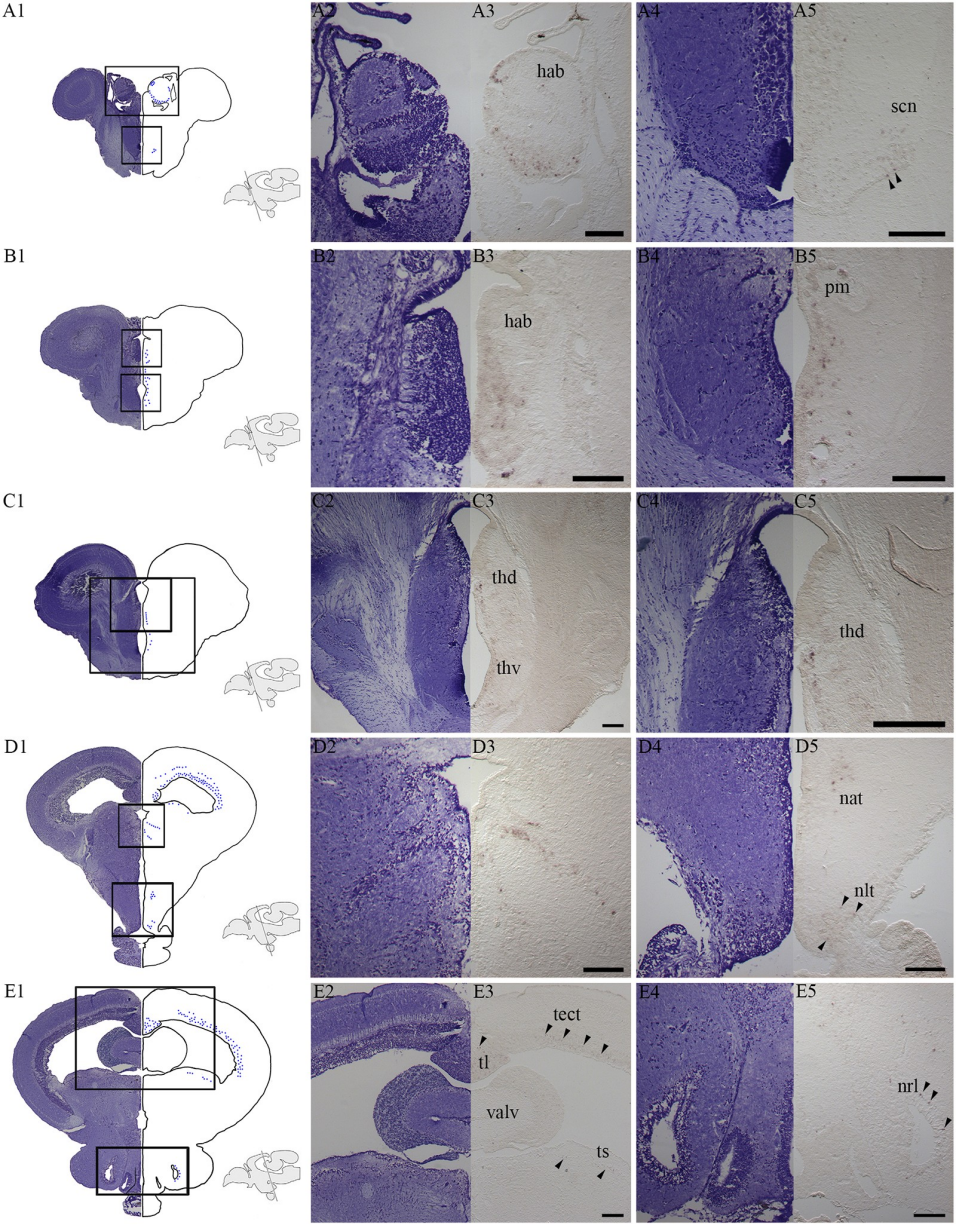
Activated brain regions after stimulation with white light for 120 minutes. **A1-E1:** Nissl-stained transverse sections at the equivalent level of *c-fos* expressing cells illustrated by blue dots in salmon, parr. Schematic drawings illustrate the level of the section. **A2-E2, A4-E4:** The Nissl-stained cell populations of interest with a higher magnification. **A3-E3, A5-E5**: *c-fos* expression at the same level and with the same high magnification. The overview illustrations (A1-E1) and the left sided tissues with *c-fos* expression (A3-E3, A5-E5) are presented by flipping the pictures, resembling a whole brain section. **A3**: Expression in a dorsal ring in the left habenula (hab) and in ventral parts of the habenula. **A5:** *c-fos* expression in the suprachiasmatic nucleus (scn). **B3:** A cell group expressing *c-fos* just ventral to the caudal habenula. **B5**: Expression in cells close to the third ventricle and in the magnocellular preoptic nucleus (pm). **C3:** Activated cells in the dorsal thalamus (thd) and ventral thalamus (thv) (posterior nucleus of thalamus) close to the third ventricle. **C5**: Focus on the expression in dorsal thalamus (thd) where the expression was strongest. **D3:** Expression of *c-fos* in caudal parts of the thalamus. **D5:** In the hypothalamus, expression was seen in the anterior tuberal nucleus (nat) and lateral tuberal nucleus (nlt). **E3:** Cells expressing *c-fos* were also localized in the mesencephalic tectum (tect), longitudinal torus (tl) and semicircular torus (ts). **E5:** Expression in the nucleus of the lateral recess (nrl). Arrowheads indicate positive cells. Scale bars of 200 μm.

Stimulation with white light (on-response, dark to light) showed *c-fos* expression in different brain regions (Fig 3). In the diencephalon, a characteristic ring of *c-fos* positive cells was located in dorsal parts of the left habenula (Fig 3A3). In addition, ventral parts of the habenula (Fig 3A3) and the suprachiasmatic nucleus (SCN) (Fig 3A5) also expressed *c-fos*. Fig 3B3 shows a cell group expressing *c-fos* just ventral to the caudal parts of the habenula and in the same section *c-fos* positive cells can be seen in cells adjacent to the third ventricle including the magnocellular preoptic nucleus (Fig 3B5). Activated cells were also detected in both dorsal and ventral parts of the thalamus (posterior nucleus of thalamus) close to the third ventricle (Fig 3C3 and 3C5). In more caudal parts of thalamus, a characteristic pattern of *c-fos* expression was observed (Fig 3D3) and in the hypothalamus, *c-fos* expression was located both in the anterior tuberal nucleus and in the lateral tuberal nucleus (nucleus lateralis tuberis (NLT)) (Fig 3D5). Positive cells have also been localized in the mesencephalic tectum, the longitudinal torus, the semicircular torus (Fig 3E3 and S1 Fig) and the nucleus of the lateral recess (Fig 3E5). Off-response in white light (light to dark) was also studied, resulting in *c-fos* expression in the same brain regions as for the on-response, although expression was in general weaker. The *c-fos* expression after stimulaton (White/Dark) was stronger than in the control (White/White) (See S2 and S3 Figs).

### Narrow bandwidth light neural activation

**Fig 4:**
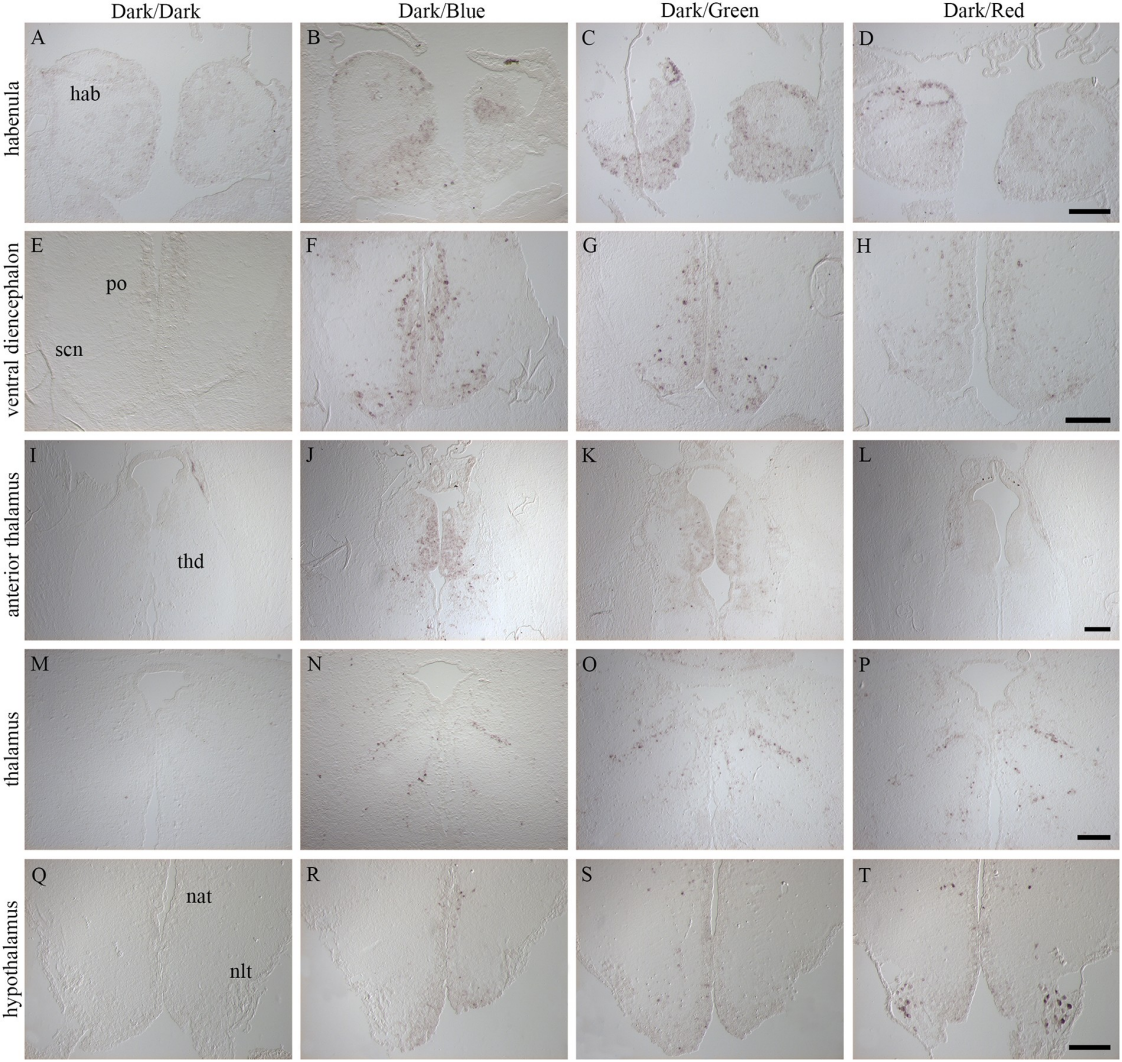
Comparison of *c-fos* positive signal in the brain of fish exposed to different narrow bandwidth light. Schematic drawings illustrate the plane of sections. **A, E, I, M, Q, U:** Control fish kept in darkness (Dark/Dark) showed no or little *c-fos* expression. **B-D:** A dorsal ring of *c-fos* positive cells was detected in the left habenula (hab) for all three spectra. **F-H:** In the ventral diencephalon, both the preoptic area (po) and superchiasmatic nucleus (scn) displayed *c-fos* expression. **J-L:** Expression of *c-fos* in the caudal habenula and dorsal thalamus (thd) for Blue (J) and Green (K), little expression in Red (L). **N-P:** Activated cells in caudal parts of the thalamus for all three spectra tested. **R-T:** In the hypothalamus, expression was detected in the anterior tuberal nucleus (nat) for the three spectra. In addition, a strong expression was detected in the lateral tuberal nucleus (nlt) for fish exposed to red light (T). Scale bars of 200 μm.

Dark-adapted fish were stimulated for 120 min with narrow bandwidth LED-light (Dark/Blue, Dark/Green and Dark/Red) and *c-fos* expression was compared against a dark control (Dark/Dark) (Fig 4). Brains from control fish kept in darkness showed no or little *c-fos* expression (Fig 4A, 4E, 4I, 4M and 4Q). In the left habenula (Fig 4B–4D), the ring of *c-fos* positive cells was detected in fish exposed to all three light spectra. In the ventral left habenula and right habenula of fishes exposed to Blue and Green light (Fig 4B and 4C), the *c-fos* expression is more prominent than in fishes exposed to Red light (Fig 4D). In the ventral diencephalon, *c-fos* expression was detected in the preoptic area and in the SCN for all spectra (Fig 4F–4H) however, the expression is stronger in fishes exposed to Blue and Green light. In the anterior part of the thalamus, expression was detected in the caudal habenula and dorsal thalamus for fish exposed to Blue (Fig 4J) and Green light (Fig 4K), while little expression was detected for Red (Fig 4L). In more caudal parts of the thalamus, the same characteristic expression pattern was observed for all spectra (Fig 4N–4P). In the hypothalamus, expression of *c-fos* was detected in the anterior tuberal nucleus for all three spectra (Fig 4R–4T) and in fish exposed red light, strong expression was also seen in the NLT (Fig 4T). Off-response in Blue, Green and Red light (light to dark) resulted *in c-fos* expression in the same brain regions as for the on-response but were in general weaker. In the habenula ring and in the anterior part of the thalamus, little or no staining was detected, and little expression was seen in the lateral cells of NLT for Red/Dark. The *c-fos* expression after stimulation (Blue/Dark, Green/dark, Red/Dark) was stronger than in the control (Blue/Blue, Green/Green, Red/Red) (See S2 and S3 Figs).

### Neural activation and deep brain photoreception

**Fig 5:**
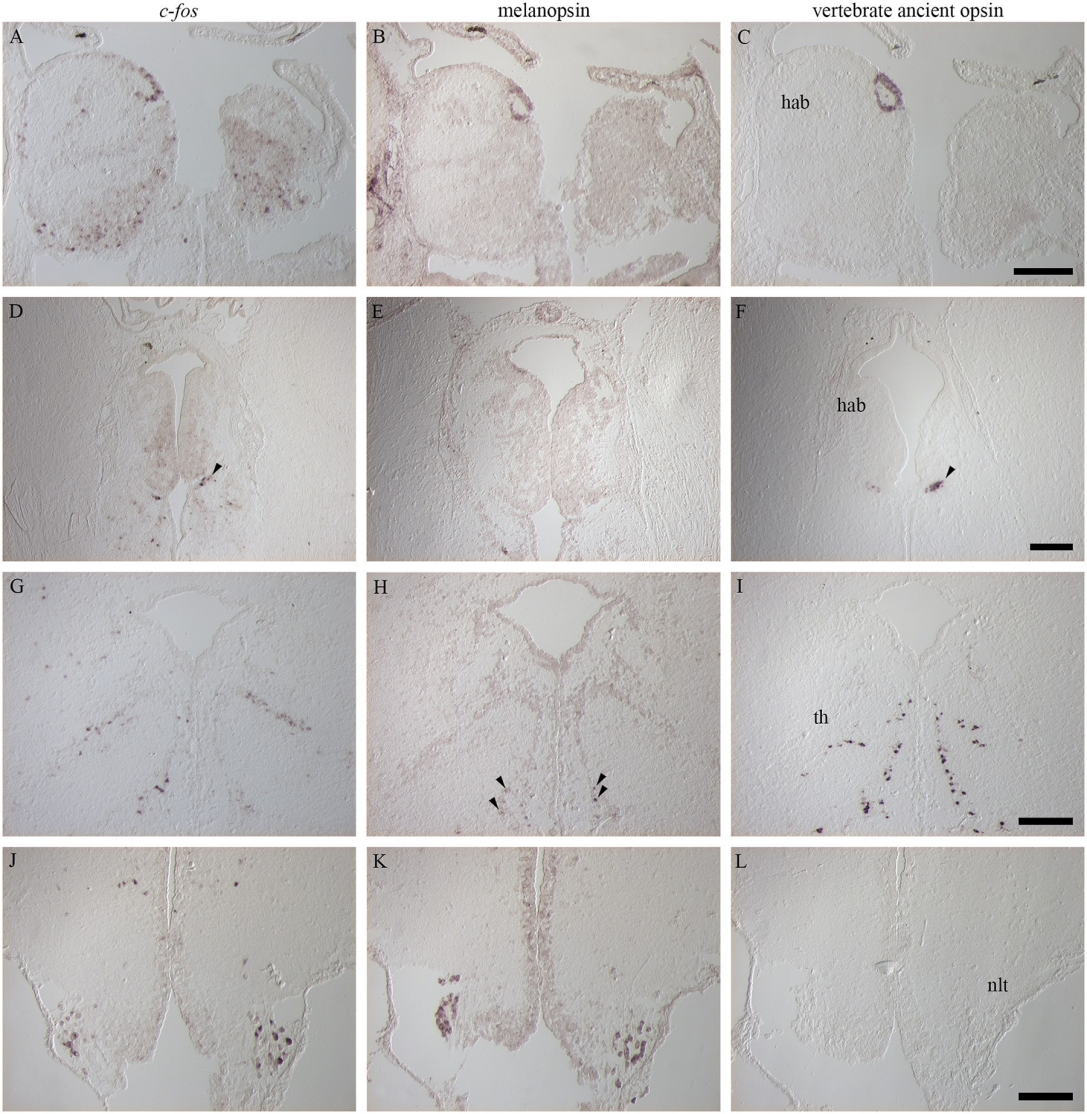
Comparison between activated brain regions, melanopsin and vertebrate ancient opsin (VA opsin) expression following exposure to different narrow bandwidth light treatments. **A-C:** In the left habenula (hab) a similar ring of cells was seen for *c-fos* (A), melanopsin (B) and VA opsin (C). **D-F:** Ventral of the caudal habenula, a small cluster of cells was detected for *c-fos* (D) and VA opsin (F) (see arrowheads) but not for melanopsin (E). **G-I:** In the caudal thalamus (th), a similar expression pattern was seen for *c-fos* (G) and VA opsin (I) and some melanopsin positive cells (H) (see arrowheads) were also detected. **J-L:** In the hypothalamus, the lateral cells of the lateral tuberal nucleus (nlt) expressed *c-fos* (J) after light activation and also melanopsin (K) but not VA opsin (L). Scale bars of 200 μm.

Neural activation and deep brain photoreception were compared (Fig 5) and showed that in the dorsal part of the left habenula, the characteristic ring of *c-fos* expression (Fig 5A) was also detected for melanopsin (Fig 5B) and VA opsin (Fig 5C). A small cluster of cells just ventral to caudal parts of the habenula was also detected for *c-fos* (Fig 5D) and VA opsin (Fig 5F) but not for melanopsin (Fig 5E). In caudal parts of the thalamus, the same characteristic expression pattern was observed for *c-fos* (Fig 5G) and VA opsin (Fig 5I), spreading laterally and ventrally from the ventricle. In addition, low melanopsin signal was detected in the same brain region (Fig 5H), even though the melanopsin expression was limited to the ventral parts. In the hypothalamus, positive cells for both *c-fos* (Fig 5J) and melanopsin (Fig 5K), but not VA opsin (Fig 5L), was localized in the NLT.

## Discussion

In the present study we showed robust *c-fos* expression in distinct cells of the Atlantic salmon brain after light stimulation that were not seen in fish kept in darkness. The cells are located in brain regions with known photoreceptive capacity that also have innervations from the retina and pineal, indicating that the neural activation can be a result of direct photoreception by deep brain cells or a combination with the retina and pineal. Though narrow bandwidth light stimulation, we showed that red light strongly activated lateral cells of the lateral tuberal nucleus.

### Time course of *c-fos* activation

Expression of *c-fos* is known to be activated rapidly and transiently as a response to a variety of stimuli. As reviewed in Kovács (2008), it is generally thought that *c-fos* and its protein are reliable markers for identifying activated cells and central nervous system circuits that respond to physiological (e.g., daily rhythm, sleep⁄wake cycle, oestrus, mating, lactation), environmental (e.g., light noise, predator odor), pharmacological and various stress challenges. Generally, the kinetics of the response to acute stimuli is characterized by a peak of *c-fos* mRNA after approximately 30 minutes and for protein after 90 to 120 minutes [31]. In accordance with this, mating stimuli in medaka (*Oryzias latipes*) and pharmacological stimuli in goldfish (*Carassius auratus*) showed a peak in mRNA level of *c-fos* after 30 minutes exposure to the stimuli [32, 33]. The time response for *c-fos* mRNA activation in the present study following light exposure revealed that a good sampling point for getting a strong *c-fos* positive signal in Atlantic salmon parr was 120 minutes. However, a weak *c-fos* stimulation was detected after 30 minutes. A similar time course has been described in rainbow trout (*Oncorhynchus mykiss*) after intraperitoneal administration of kainic acid [34] where *c-fos* mRNA was detectable after only 30 minutes but reached a peak after 120 minutes. The delay in elevated *c-fos* expression observed in the present study may be related to temperature (temperate fish species vs. tropical) and/or the type of stimulation as a study in Atlantic salmon has shown strong *c-fos* expression after acute stress for 30 minutes [35].

### Light induced neural activation of the brain

Previous studies performed in adult zebrafish showed that *c-fos* mRNA expression was induced in specific and discrete brain regions following a 30 minutes light pulse applied at night [20]. Increased gene expression levels of *c-fos* were detected mainly in the pretectum, suprachiasmatic nucleus, tectum, longitudinal torus, valvula cerebelli, and hypothalamus. In comparison, a study in zebrafish larvae using a combination of zebrafish atlas and immunohistochemical detection of phosphorylated-extracellular signal-regulated kinase as a readout of neural activity, showed activation in subpalladium, tectal neuropil, cerebellum, and hindbrain after a 10 second light pulse. Further, blue light stimulation showed increased activity in vestibular nuclei, habenula, hypothalamus, ventral hindbrain and spinal cord [36]. The present study confirmed activation of distinct and specific brain regions in Atlantic salmon stimulated with light for 120 minutes that were not seen in fish kept in darkness. These regions included the habenula, suprachiasmatic nucleus, thalamus, hypothalamus, tectum, longitudinal torus andsemicircular torus. Importantly, several of these brain regions also contain deep brain photoreceptors, expressing melanopsin and vertebrate ancient opsin, suggesting a potential direct activation of these regions upon light stimulation. However, several of the brain regions expressing *c-fos*, have retinal and pineal innervations [1] and we cannot exclude that the cells may also be activated as a response to photoreception in the retina or pineal, or in combination. The present results showed no *c-fos* expression in the pineal organ (see S4 Fig) even though the fish pineal organ is known to contain a photoreceptive circadian oscillator [37].

### Habenula

A characteristic dorsal ring of the left habenula showed a similar expression pattern for *c-fos*, melanopsin and VA opsin, and studies by Sandbakken et al. (2012) have shown that the *Xenopus*-like melanopsin (*Opn4x)* cell population in the habenula is co-localized with an asymmetric serotonergic cell group of the left habenula [38]. In salmonids, the left habenula is shown to receive the majority of the innervations from the parapineal organ and the flattened terminal field of the parapineal tract is shown to be located in the dorsal part [39]. Photoreceptors of the left habenula could be related to the assumed photoreceptive function of the parapineal organ as suggested by Sandbakken et al. (2012) that could have a functional relationship also to the limbic system [39]. Interestingly, the neural activity of habenula seen in zebrafish larvae using the phosphorylated-extracellular signal-regulated kinase as a readout of neural activity, is suggested to be linked to a putative visual network, however the activation was seen in the whole left habenula and not in a dorsal ring [36]. Further, using light-sheet microscopy to record activity, reported through the genetically encoded calcium indicator GCaMP5G, showed activity in the left dorsal habenula in zebrafish larvae after red LED flash stimulation. This activity was associated with a visual response linked to the dorsal interpeduncular nuclei of the midbrain that failed to show robust responses in dorsal cells of left habenula after ablation of the eyes [40]. Zebrafish seem to lack the dorsal ring of nonvisual opsins in the left habenula that we find in salmon. Although, we cannot exclude that the neural activation of the dorsal ring of the habenula in salmon is caused by the innervations from retina or pineal, the overlap between melanopsin, VA opsin and *c-fos* in the distinct ring, supports the idea of a direct neural activation in these photoreceptive cells. Such overlap was also seen in our study in Atlantic halibut, showing *c-fos* activation in the dual photoreceptive hindbrain cluster expressing both VA opsin and melanopsin [13].

### Suprachiasmatic nucleus (SCN)

The SCN is considered to be an integration area for photic information in the teleost brain, receiving both retinal and pineal input [41]. The SCN is also a major source for dopaminergic innervations of the pituitary in Atlantic salmon [42]. It has been shown that *Xenopus*-like melanopsin positive cells (*Opn4x)* in the SCN are co-localized with a dopaminergic cell population and it has been suggested that the melanopsin positive cells in the SCN have a role in the dopaminergic regulation of the pituitary function [12]. In accordance with zebrafish exposed to a 30 minutes light pulse at night [20], results from the present study confirmed that this important integration area is also activated in salmon following stimulation with light. The neural activation might be a result of direct photoreception or a combination of activation from the retina and pineal.

### Thalamus

In adult zebrafish, the VA opsin positive cells of the thalamus appear from the anterior thalamic nucleus just ventral to the habenula and spread caudally and laterally to the intercalated thalamic nuclei. The VA opsin positive cells have been shown to all be GABAergic as *gad67* mRNA co-localize with *valop* mRNA and it has been suggested that this thalamic population may regulate light-avoidance behavior in zebrafish [10]. Moreover, the thalamic VA opsin population in zebrafish has diurnal rhythmicity and it has been suggested that the thalamic cell group maximizes its photosensitivity diurnally to regulate neuronal activity in the thalamus during dawn and dusk [43]. The thalamic population of VA opsin positive cells in salmon has been shown to have a similar pattern spanning caudally and laterally from the sub-habenula region terminating at the level of the posterior commissure [44]. The present study demonstrated that *c-fos* is expressed after stimulation with light in the same thalamic regions as VA opsin e.g., anterior thalamus just ventral to the caudal habenula and in posterior parts where the VA opsin positive cells are spread caudally and laterally, although we cannot conclude that the expression is in the same cells. In the caudal thalamus, melanopsin positive cells are also apparent laterally, these are shown to be the mammalian-like (*Opn4m)* by Sandbakken et al. (2012) revealing both *Xenopus*-like and mammalian-like melanopsins to be apparent in brain regions with neural activation. In zebrafish, the deep brain VA opsin neurons have been suggested to have a role in time- and light-dependent physiology to adjust to environmental changes [43] and it can therefore be suggested that activated thalamic population in salmon has a similar role. Thalamus is known to be innervated by both the retina and pineal [1] and the neural activation might be a result of direct photoreception or a combination of activation from the retina and pineal.

### Lateral cells of the lateral tuberal nucleus (NLT) show neural activation in red light

The hypothalamus is known to be the major source of innervation in the pituitary of teleosts [45–47] and present results revealed that cells in the NLT are activated after light stimulation. Interestingly, lateral cells of the NLT known to express mammalian-like (*Opn4m*) melanopsin in salmon [12] were activated in dark-adapted salmon stimulated with red light. The neuroendocrine regulation of many of these lateral neurons expressing melanopsin suggests a role in modulating pituitary hormone release and function [12]. Studies in rainbow trout, sea bass (*Dicentrarchus labrax*) and salmon have shown that the relative transmission of light through the cranium is dependent on the spectral content and that there is a higher penetration towards the red end of the spectrum [48, 49]. Higher transmission of red light through the cranium may explain the presence of photoreceptors detecting red light in the hypothalamus, deep in the brain. Notably, in the present study we have standardized the intensities between light spectra, but wavelength specific differences in cranial absorbance may have resulted in different exposure of light intensity in the brain and pineal. Both the aquatic environment and tissue act as potent filters of the light significantly modifying spectrum and intensity, highlighting the difficulty to dissect light effects in fish. Furthermore, red light has been shown to be less efficient in salmon on suppressing nocturnal melatonin than blue and green light, as the melatonin levels were shown to increase under high intensity of red light reaching 40% of night levels [50]. The light intensity required to suppress diel melatonin production in salmon is higher than for sea bass and cod [49, 50] and both light intensity and spectral composition must be evaluated considering the photoneuroendocrine regulation of the salmon lifecycle. However, the light intensity used in this study is well above the light intensity threshold for suppression of melatonin in Atlantic salmon pineal [49] while the spectral properties of nonvisual opsins in salmon is limited to VA opsin with a λmax of 451 nm [51]. In zebrafish, members of the O*pn4m* and *Opn4x* class were shown to have a λmax of 484 nm and 470 nm, respectively [52] and a similar λmax for melanopsin in salmon is likely, contradicting the presumable neural activation of melanopsin positive cells in the NLT. A potential co-expression of a long-wavelength light-sensitive opsin with the melanopsin positive cells of the NLT might be suggested.

In conclusion, this study of light activated neurons in the Atlantic salmon brain, indicates light stimulation of deep brain photoreceptors, by distinct expression of *c-fos* in photoreceptive brain regions.

## Supporting information

S1 Fig Details on Fig 3E3. (A) Overview of the section with boxes indicating the sections of B-D. (B) Arrowheads indicates c-fos expression in the tectum and in the longitudinal torus. (C) Arrowheads indicates c-fos expression in the semisircularus torus. (D) Higher magnification of some of the cells in C. Scale bars 200 μm.
(TIF)

S2 Fig Off-response stimulated (light to dark). (A, E, I, M, Q) White/Dark, (B, F, J, N, R) Blue/Dark, (C, G, K, O, S) Green/Dark, (D, H, L, P, T) Red/Dark. In general less or weaker expression of *c-fos* is detected in the off response then in the on-response. Scale bars 200 μm.
(TIF)

S3 Fig Off-response control (light to light). (A, E, I, M, Q) White/White, (B, F, J, N, R) Blue/Blue, (C, G, K, O, S) Green/Green, (D, H, L, P, T) Red/Red. In general little or weaker expression of *c-fos* is detected in the controls. Scale bars 200 μm.
(TIF)

S4 Fig Pineal organ. There are no expression of *c-fos* in Dark/White stimulated fish. Scale bar 200 μm.
(TIF)
