# Supplementary figures and images for: Neural activation in photosensitive brain regions of Atlantic salmon (Salmo salar) after light stimulation
Source: PLoS One. 2021 Sep 29;16(9):e0258007. doi: 10.1371/journal.pone.0258007 (PMC8480854; doi:10.1371/journal.pone.0258007)

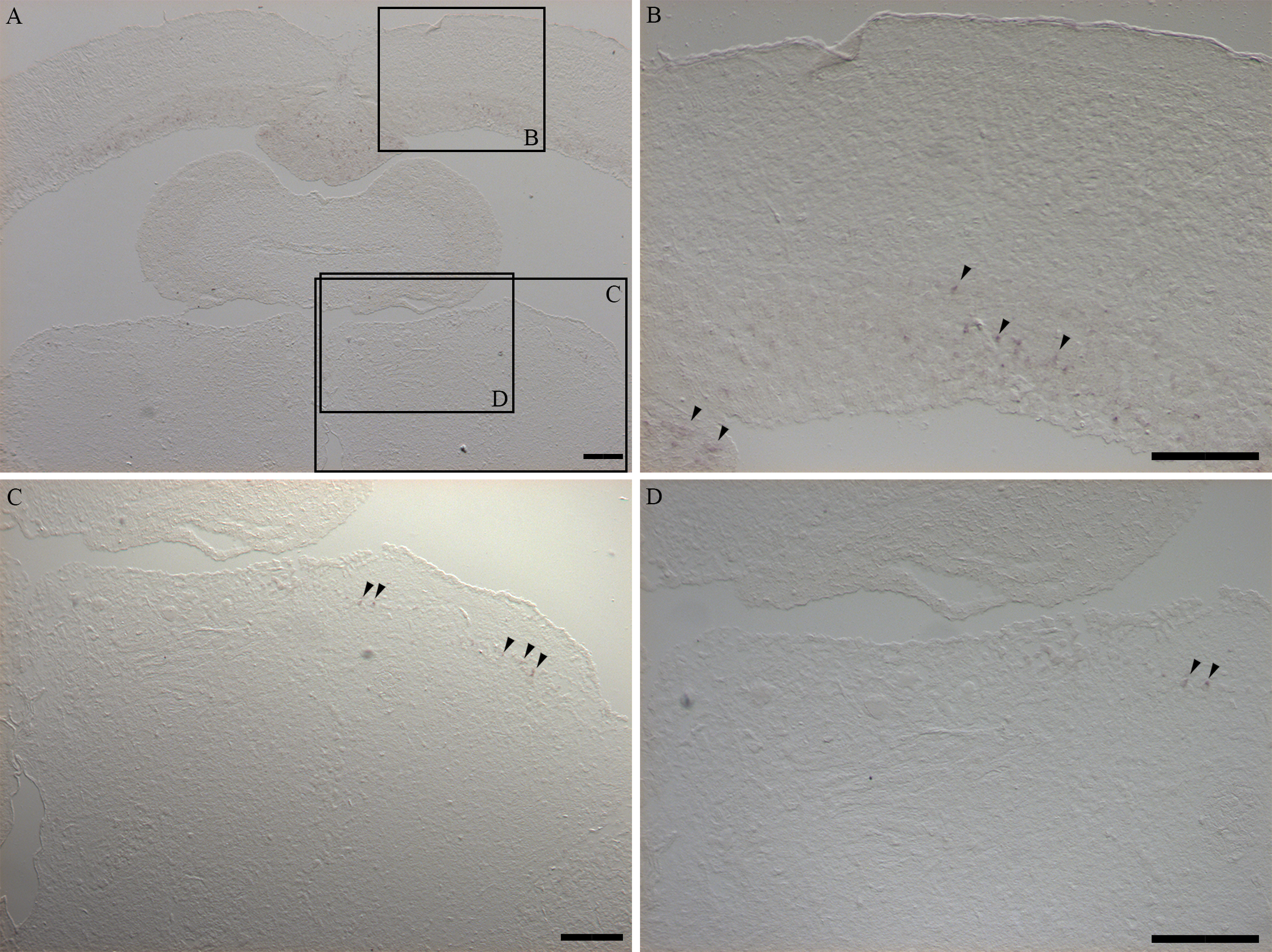

Supplement: S1 Fig — (A) Overview of the section with boxes indicating the sections of B-D. (B) Arrowheads indicates c-fos expression in the tectum and in the longitudinal torus. (C) Arrowheads indicates c-fos expression in the semisircularus torus. (D) Higher magnification of some of the cells in C. Scale bars 200 μm. (TIF) [file pone.0258007.s001.tif]

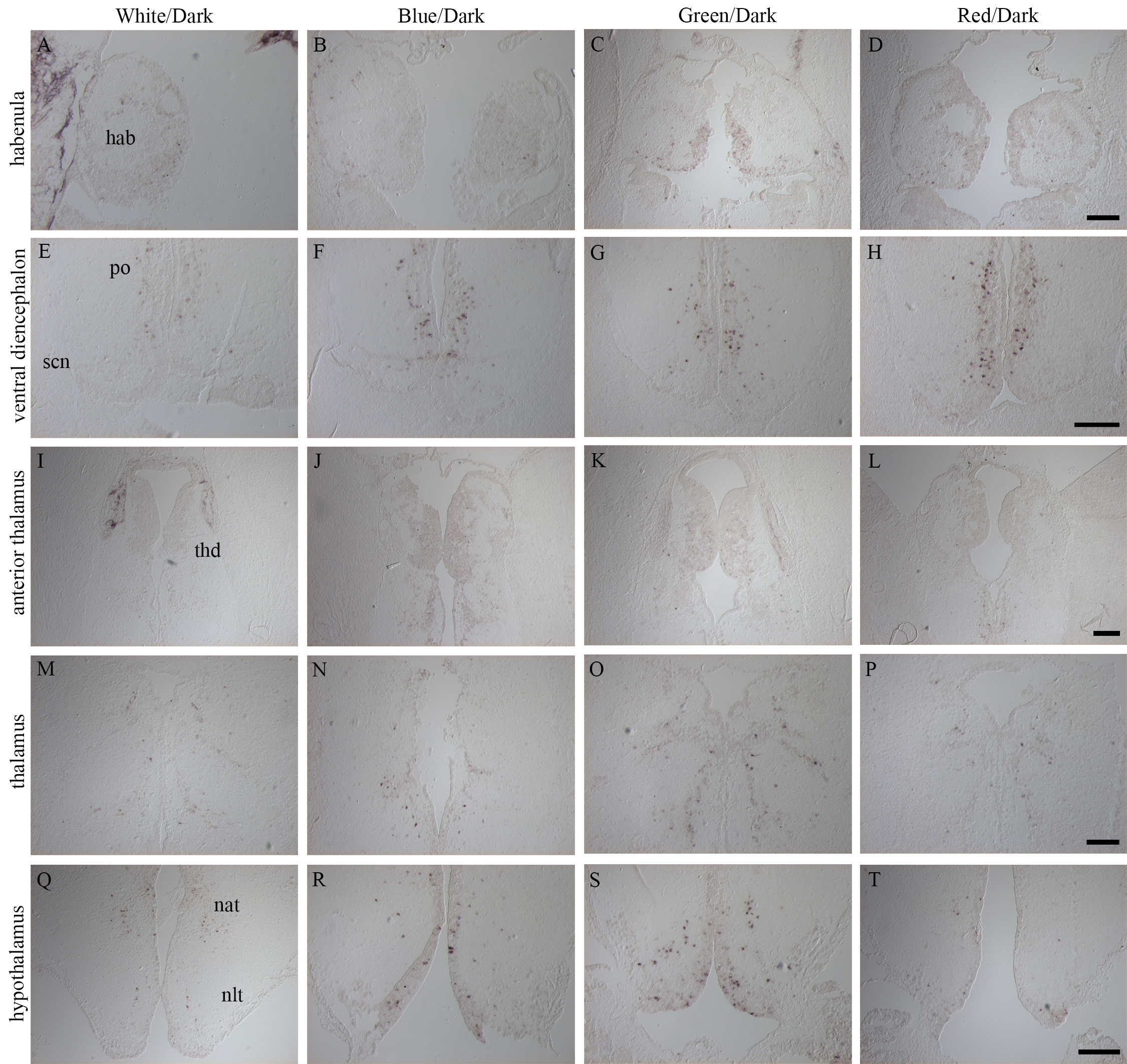

Supplement: S2 Fig — (A, E, I, M, Q) White/Dark, (B, F, J, N, R) Blue/Dark, (C, G, K, O, S) Green/Dark, (D, H, L, P, T) Red/Dark. In general less or weaker expression of c-fos is detected in the off response then in the on-response. Scale bars 200 μm. (TIF) [file pone.0258007.s002.tif]

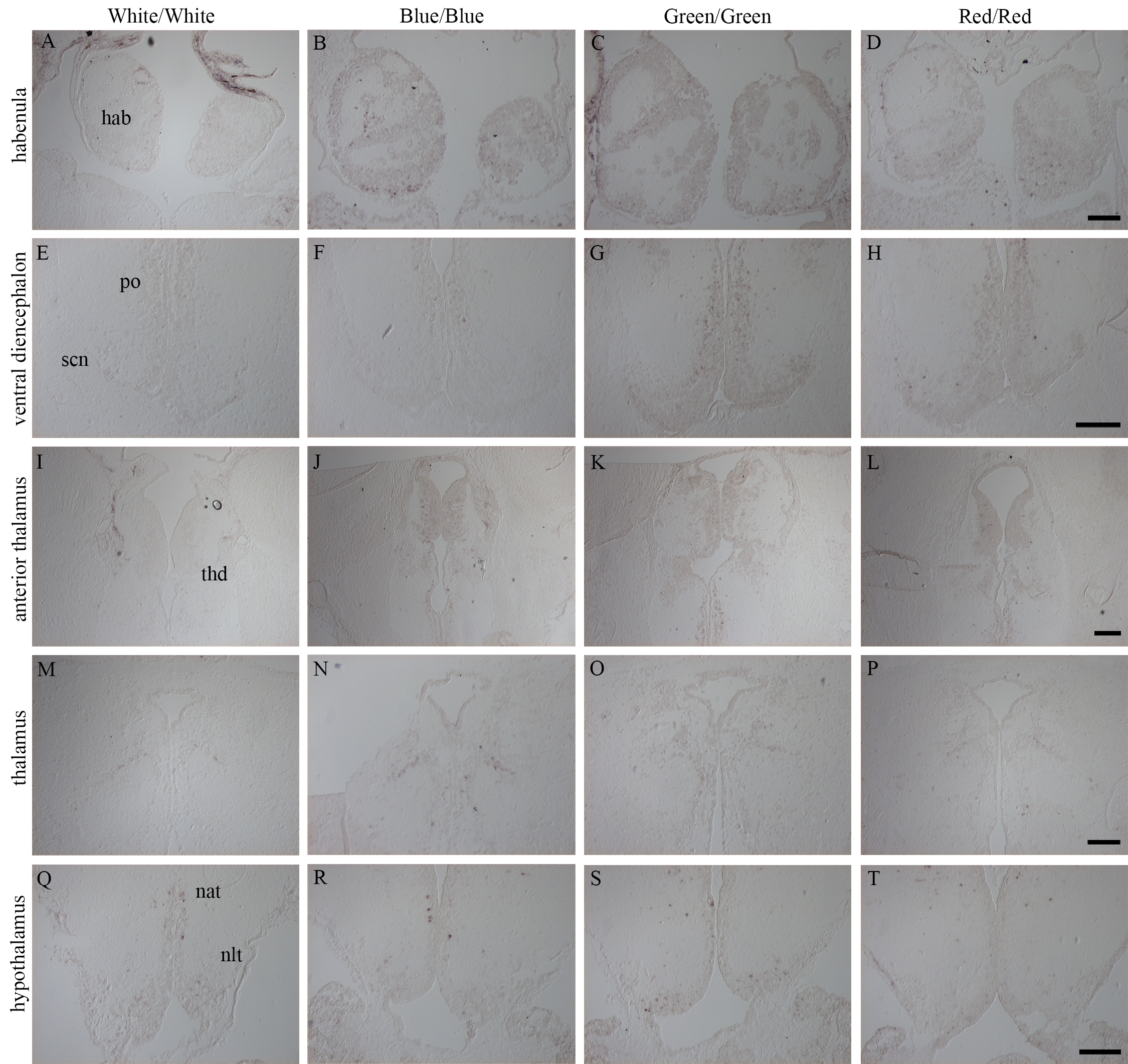

Supplement: S3 Fig — (A, E, I, M, Q) White/White, (B, F, J, N, R) Blue/Blue, (C, G, K, O, S) Green/Green, (D, H, L, P, T) Red/Red. In general little or weaker expression of c-fos is detected in the controls. Scale bars 200 μm. (TIF) [file pone.0258007.s003.tif]

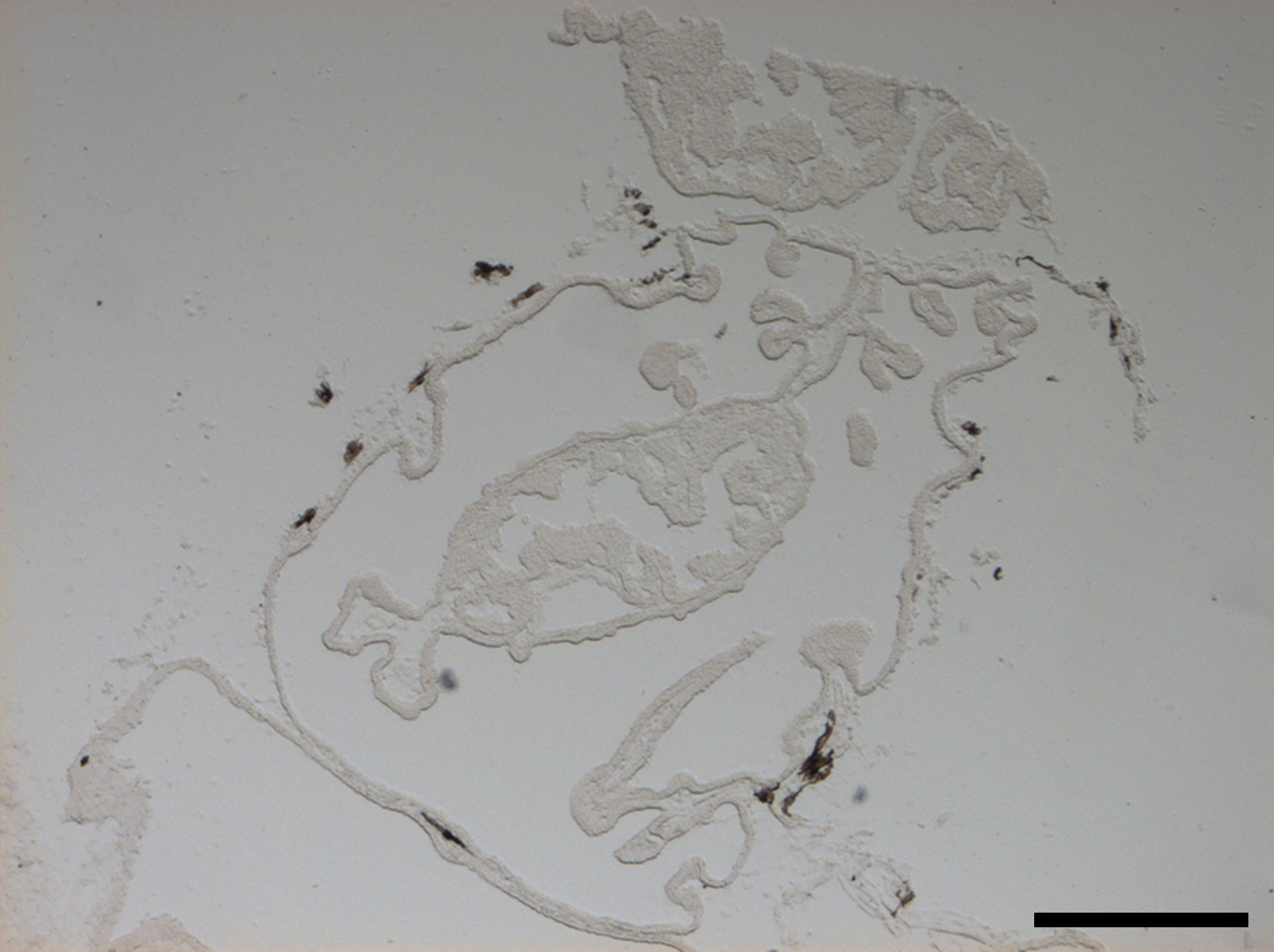

Supplement: S4 Fig — There are no expression of c-fos in Dark/White stimulated fish. Scale bar 200 μm. (TIF) [file pone.0258007.s004.tif]
